# Sustainable Environmental Strategies for Shrinking Cities Based on Processing Successful Case Studies Facing Decline Using a Decision-Support System

**DOI:** 10.3390/ijerph16193727

**Published:** 2019-10-03

**Authors:** Francisco Sergio Campos-Sánchez, Rafael Reinoso-Bellido, Francisco Javier Abarca-Álvarez

**Affiliations:** Department of Urban and Spatial Planning, University of Granada, Granada 18071, Spain; rafaelreinoso@ugr.es (R.R.-B.);

**Keywords:** shrinking cities, environmental planning, territorial sustainability, analytic hierarchy process, decision-support systems

## Abstract

Since the middle of the last century post-industrial cities around the world have been losing population and shrinking due to the decline of their structural growth models, showing important socioeconomic transformations. This is a negative phenomenon but one that cities can benefit from. The aim of this work is to verify what type of measures against urban decline would be most suitable if applied to a specific case study. To do this, international cases of shrinking cities where successful measures were already carried out facing decline: (i) are collected, (ii) are classified based on several influencing criteria, and (iii) are grouped under similar alternatives against the decline. Measures and criteria focused on achieving sustainability are emphasized. Alternatives are then prioritised using an Analytic Hierarchy Process designed at several hierarchical levels. The results are discussed based on the construction of sustainable future scenarios according to the optimal alternatives regarding the case study, improving the model validity. The work evidences that environmental and low-cost measures encouraging the economy and increasing the quality of life, regardless of the city size-population range where they were performed, may be the most replicable. Future research lines on the integration of the method together with other decision-support systems and techniques are provided.

## 1. Introduction

The Shrinking Cities International Research Network (SCiRN)—a worldwide research consortium of scholars and experts from various institutions (30 members from 14 countries) pursuing research on shrinking cities in a global context to advance international understanding about population decrease and urban decline as well as causes, manifestations, and effectiveness of policies and planning initiatives so stave off decline—, agreed in 2004 that the term ‘shrinking city’ (i.e., declining cities) refers to a global phenomenon by which, since the middle of the last century, several hundred cities and urban areas (> 10,000 inhabitants—in 370 cities with more than 100,000 residents—) have been losing population, while undergoing major economic transformations, evidencing the decline of their structural growth models. This is mainly due to the lack of competitiveness of their manufacturing industrial production model, to which other facts contribute, e.g., suburbanization, war, natural disasters, ageing population, low fertility, or socialist systems breakdown [1,2]. Pallagst et al. introduced multidimensionality as an additional attribute of this phenomenon, adding social decline and environmental impacts to economic problems [3]. Today, many cities and regions are still trying to address this problem using different recovery strategies.

The urban decline varies according to territories, continents and particular casuistry. In Europe this problem was/is traditionally due to low fertility, emigration, the real estate boom, housing abandonment or de-industrialization, which has led to the testing of recovery strategies of diverse amplitude, profile and cost. Among others, some examples of these are those focusing on restoring market balance through housing demolitions (e.g., Germany in the 1990s), urban regeneration (e.g., in U.K.), or attracting foreign investment and increasing territorial competitiveness (e.g., in post-socialist countries) [4]. In Asia the reasons for shrinkage had/have more to do with the population ageing, exodus from rural areas to large metropolises, property speculation or economic inequality (e.g., in Japan, and in the near future in China). Since the 1970s, in North America these problems are based on suburban expansion and deterioration without regional planning perspectives, or on national economic restructuring. In Latin America they had/have to do with socio-economic inequality, emigration or the population displacement to large metropolitan regions, for example [3,5,6].

### 1.1. The Spanish Case

The shrinkage phenomenon was not as important in Spain as in other territories within the European context, due partly to Spain’s late incorporation into the industrial process and the characteristics of its economy, which is more focused on services and tourism [7]. However, the causes and effects of shrinkage are similar to those of other countries. From the 1970s onwards, the end of industrial-mining economic and productive activity in highly specialised and resourceful areas went into decline, losing population and threatening their economic and social structures [8].

Among the few initiatives facing the decline in Spain, those developed in the Spanish (industrialised) north stand out. Perhaps because of its relevance, the most paradigmatic example is probably the deep industrial transformation of the Bilbao’s waterfront from the end of the last century, involving important urban interventions (e.g., Bilbao Ria 2000 project) that would carry the name of “Guggenheim effect” [9]. Also relevant are the cases of some significant Spanish mining and industrial cities, such as (i) the partial recovery of Ponferrada, thanks to industrial investments; and (ii) to a lesser extent, Puertollano, due to its renewable energy factories and its trend towards green tourism and the reduction of CO_2_; or the socio-economic regression process of some mining cities of Asturias (e.g., Mieres, Langreo) until today [10].

Similar processes (adjustment, reindustrialization, outsourcing, and urban transformation) took place in all of them, but with different results [10]. One of the most noteworthy cases was the post-industrial (after steel and iron industry), functional and landscape transformation of Avilés by urban tourism. The measures focused on the use of brownfields, environmental decontamination, regeneration of the port, historic centre improvement, and tourism policy, with important projects such as the building of the Niemeyer’s Cultural Centre, which was not without management and financial problems [11]. In many of these cases, urban transformations involved parallel restoration and reuse of industrial heritage [12].

Currently, the shrinkage in the northern Spain and other areas of this country are still active. There is evidence of this, such as the increase in company closures and unemployed number, e.g. the steel companies Alcoa Company in La Coruña and Avilés, and Megasa Company in Ferrol, the naval one in Sestao, or the wind turbine plant of Vestas Company in León. Structural reasons of a productive and technological nature, lack of competitiveness and rising electricity prices are argued. To this is added the current transition of the European Union (EU) towards an economy free of greenhouse gases and the compliance with the Paris Agreement against the climate change, which lead to the closure of all non-competitive thermal and coal production plants by 2030. This has an impact on those European territories where coal still supports employment, such as Spain. Specifically, EU’s decision 787 ordering the immediate closure of all non-competitive mining activity by the end of 2018, currently affects several mining companies and cities in northern Spain, such as the case of Hunosa Company in Mieres, reported in the press.

### 1.2. Starting Point

The working hypothesis is that specific solutions against urban decline successfully developed in certain territories could be as suitable for others, depending on the similarity and potential of each context. The lessons learned from this type of successful experiences would be useful as recovery strategic models for other cities that have not yet taken the appropriate measures or in which the measures developed have not had the expected success. This would be as long as the appropriate comparative framework was provided based on the context of both the successful urban decline experiences and the specific case study.

This would be done by answering the following research questions: Does decision-making help to explore the suitability of multiple effective alternatives against the shrinkage? What types of initiatives carried out more or less effectively in decline cities would be most likely to be replicated successfully in other similar case studies? What factors would be the most important to manage taking into account contemporary aspects such as investment priority of sustainability? The challenge is to answer these questions by analysing case studies using a hierarchical decision model, which could serve as a guide for planning professionals facing urban decline problems.

The aim of this work is to explore successful city cases against urban decline using a decision support system to prioritize lessons learned in terms of sustainability, what is done regarding a specific case study. This exploration is carried out in a comparative and integrated way at a macro level. The subsequent discussion of the results according to the potential of the case study to replicate the optimal measures against decline details the model applied. In addition, the integration suitability of the analysis method developed together with other analysis techniques is emphasized. The work is relevant as a useful method to select initially those measures facing urban decline that would best suit a specific case study, fitting as a guiding instrument within sustainable planning. 

## 2. Research Background

### 2.1. Individual Case Studies VS Integrated Comparative Studies

Given this shrinking cities global scenario, since the beginning of this century, many programs and meetings were held, especially to expose the various cases of decline and to make this problem known to the international community. Some of the best known may be the Shrinking Cities project in the 2000s [13], the Shrinking Cities International Research Network (SCiRN) in 2004 supported by the Institute of Urban and Regional Development at the University of California (Berkeley), the COST-Action CIRES programme, or the Shrink Smart project. All of them have brought together academics, professionals and experts who researched the paths and results obtained in different cities. 

However, it seems that this diversity of initiatives often resulted in international debates based on the exposure of individual local cases without agreement on common research strategies on the phenomenon. In contrast, comparative research between case studies should better enable the influence of local conditions to be isolated and criteria to be unified. In addition to large-scale quantitative research on urban decline, comparative case studies provide valuable information on specific patterns and paths of decline [14]. Extensive international comparative studies have helped to highlight the relevance of shrinkage [8,15]. Within this group of studies, some of them, such as that of Großmann et al. (2013) support more integrated research focused on removing transnational knowledge barriers [14]. 

At the UC Berkeley symposium in 2007 organized by the Centre for Global Metropolitan Studies together with the Institute of Urban and Regional Development, SCiRN-sponsored research made it clear that the comparative approach between this type of cities by studying cases from a global perspective is unique and innovative in improving the quality of life in them. Nevertheless, there is still little comparative work that collects the lesson learned in each case in an integrated way [5], which could be useful to the planning and political agenda of the involved cities.

An example of this last group of works is the research by Sánchez-Moral et al. focused on Avilés (Asturias, Spain), close to the case study of this work [16]. The strategies developed in the 2000s to revitalise the economy of Avilés included measures already taken in other manufacturing regions of Europe, e.g., privatisation of public companies, attraction of foreign investment, reorientation of the economy towards innovation and creativity, or development of iconic projects, such as Niemeyer Centre.

### 2.2. Studies on Urban Decline within the Sustainability Framework

The urban decline is generally something negative for a city, but it can also be used as a solution to the problem, for example from an ecological point of view: space available for other post-industrial opportunities, free land once it has been decontaminated, reuse of old industrial buildings, fresh air, etc. In addition, the urban development opportunity that this available space could offer should be taken advantage of within the context of the 2030 Agenda for Sustainable Development Goals (SDG) of the United Nations (UN). It seems that it is time for planners and decision-makers who have traditionally favoured the growth and expansion of cities to think now about appropriately planning their shrinkage, but how to do that is an important question. 

Studies focused on finding answers against decline that, rather than local interest, support a recovery based on the common good are particularly interesting due to their emergence, such as those: (i) that are influenced by a growing awareness of climate change [17,18]; (ii) that suggest using a resilient perspective to deal with this type of change by means of an approach that combines ecology and economy (e.g., the use of forests as nature tourism) [19], or that address the urban form (e.g., by assessing urban compactness) [20]; (iii) that rely on the social dimension (capital, social mix, creative talent) and the use of endogenous resources to drive urban economic growth [21,22]; (iv) that suggest a change of paradigm through ‘smart shrinkage’ in a planned way; (v) that address the complex relationships between socio-demography, infrastructure, land-use, ecosystem services and biodiversity of shrinking cities to ensure urban quality of life and healthy urban ecosystems under shrinkage conditions [2]. This is by reviewing the principles on which traditional urban policies were based, focusing primarily on growth and expansion [5,23,24,25]. These last works analysed sustainable recovery strategies based, for example, on the depopulation of deteriorated neighbourhoods, the ecological management of the post-industrial land, the new challenge for both land-use and biodiversity research, or the use of economic development plans that emphasized controlled urban shrinkage. 

### 2.3. Assessment of Case Studies Using Decision-Support Systems

Therefore, an approach that assesses successful experiences against decline in a comparative and integrated way, and obtains relevant information from them, seems to be useful for decision-making on a specific shrinking case study. In this regard, it is recognized that Decision Support Systems (DSS) are effective tools for the incorporation, integration and decision support of complex problems by reducing indeterminacy and improvisation on their resolution [26]. Basically, decision-making consists of a selection process among alternative courses of action based on a set of criteria to achieve one or more aims [27]. The multi-criteria evaluation (MCE), defined as a set of techniques to support decision-making processes, is useful for this purpose. It is based on the weighting of variables that influence the decision activity, and that have to be previously classified for selection among alternatives. 

Urban shrinkage is a complex problem, due to the diversity of processes and stakeholders involved, so decision support can be a useful tool for analysis. There are many studies on decision support, and also on shrinking cities, but there are a few studies that work on both issues in an integrated way, hence its novelty. Some examples of the latter could be (i) the multi-criteria evaluation that Schetke & Haase carried out to assess the socio-environmental impact of the urban shrinkage suffered by many German cities of the old GDR [28]; or (ii) the prescriptive decision model (OR/MS) developed by Johnson et al. for the management of brownfields as a neighbourhood development strategy in a small town of Bristol (MA, USA) [29]. The former studied the socio-environmental impact of micro-scale demolition as a strategy against decline in several urban areas of Leipzig, showing development differences between these areas, such as urban expansion versus redensification and redevelopment. This is due to assumption of demolition as a generic strategy facing decline in this region. However, a preliminary analysis that initially took into account different strategic options could open up new ad hoc opportunities against decline in each case, depending on its features. In addition, other interesting empirical studies on shrinking cities from a sustainable environmental perspective were cited and compiled in the research of Haase [2]. 

The approach to evaluate successful initiatives against decline regarding a particular case study depends on a specific aim and process in which some alternatives are selected from a group of them to obtain the optimal one. For this reason, the Analytical Hierarchy Process (AHP), developed by Saaty, seems to be a suitable DSS for application in several problems of this type [30]. By means of comparison in pairs it is possible to identify the relative importance of each criterion and decision alternative. The AHP has already been used to weight sustainable urban decline measures. For example (i) Lee and Lim’s approach based on an AHP oriented assessment of urban regeneration projects in South Korea according to three sustainability goals (physic, social and economic) by comparing economic and community indicators [31]. This is a local study that excludes aspects such as environmental sustainability and project costs, which may lead to some biases; or (ii) the multi-criteria analysis according to AHP hierarchical modelling carried out by Hemphill et al. in the light of a literature review on sustainable urban regeneration interventions and policies and their evaluation indicators [32]. It is a broad study in its approach and addressed to an expert panel but with no specific cases of applicability. 

## 3. Materials and Methods

In general, the methodology is developed by taking the following steps: (1) Collection of case studies against urban decline and successful practices that were generally carried out in them. (2) Design of a hierarchical decision support model (AHP) by classifying the above information according to criteria (influencing factors) and alternatives (initiatives adopted). (3) Application of the AHP model by comparing criteria and alternatives, and prioritizing results. Finally, the results are discussed and future lines of research are recommended. 

### 3.1. Case Study

This work focuses on an important post-industrial mining medium-sized city in northern Spain: Mieres (Asturias). It is a highly representative case of active processes of urban decline with economic-industrial origin in this country. Mieres has a population of 38,962 inhabitants according to the Spanish Statistical Office—known in Spanish as the INE—in 2017. It was an important mining urban node during the 19th century thanks to the coal production, being also a steel and iron centre until 1970, which is evidenced by the existence of a rich industrial heritage. (e.g. coal washer El Batán, currently being exploited by the Hunosa company). The crisis in both economic sectors has led to the loss of more than 30,000 inhabitants from 1960 (when the city had a population of 70,871) to the present day. Table 1 shows a summary characterisation of the case study based on a partial strengths, weaknesses, opportunities, and threats (SWOT) analysis. 

### 3.2. Case Collection

The response of regions and cities against the decline has been and is being very diverse, depending on the particular conditions and the socio-economic and political context in each case [33]. Through a literature review, macro-level measures against urban decline that were successfully carried out in selected international cities and regions were collected and characterised. The list is not exhaustive; only the most frequently mentioned or best documented cases were collected through multiple studies and reports. The information obtained from the compilation of experiences was detailed in order to be able to compare them with a specific case study. This would allow the subsequent design of the decision-making model. Each case was characterised by the following steps (see Table 2): (i) case identification; (ii) identification of the declining socio-economic sector; (iii) source identification; (iv) macro-level description of the dominant recovery initiatives from the decline developed in each case; and (v) identification of the dominant sustainable profile that supports or feeds each initiative. 

### 3.3. Decision-Making Model Design

#### 3.3.1. Hierarchical Levels

The first level or model goal (N1) is to know which alternative is the most suitable to achieve the aim of the work. In other words, which initiative against the urban decline already tested in other cities or regions related to the specific case study (Mieres) would be more likely to succeed if applied to the latter. 

For the second hierarchical level design (N2), the case collection information is classified according to criteria (influencing factors). In the literature review, the factors that have commonly characterised the chosen sample of frequent cases (n = 25) were the following: (C1) Population-size range. A distinction is made between large, medium-size and small cities. (C2) Origin of urban decline involving population loss. Three types of main causes are identified: environmental, social or economic. (C3) Profile of the dominant initiative facing decline. As argued above, from a sustainability perspective three types of dominant profiles are also identified: environmental, social and economic. (C4) Cost of the initiative (economic, political, social, resource consumption level, etc.) by nominal categorizing the cost levels as high, medium or low. In order to simplify the model, similar cases were grouped in this hierarchical level, i.e., cases that show the same attributes according to the mentioned criteria (C1–C4). Within alternative H (8, 11, 24, i.e., Estonia/Central Germany–Ivanovo–St. Louis, respectively) it was considered that case nº 8 (Estonia and central Germany’s medium-sized and small cities) accumulates a large population as a whole, and could therefore be grouped together with cases nº 11 and 24.

The alternatives or third hierarchical level of the decision-making model (N3) were identified with the initiatives or groupings of them facing urban decline in each case, according to the cities or urban regions where they were carried out (n = 12).

**Figure 1:**
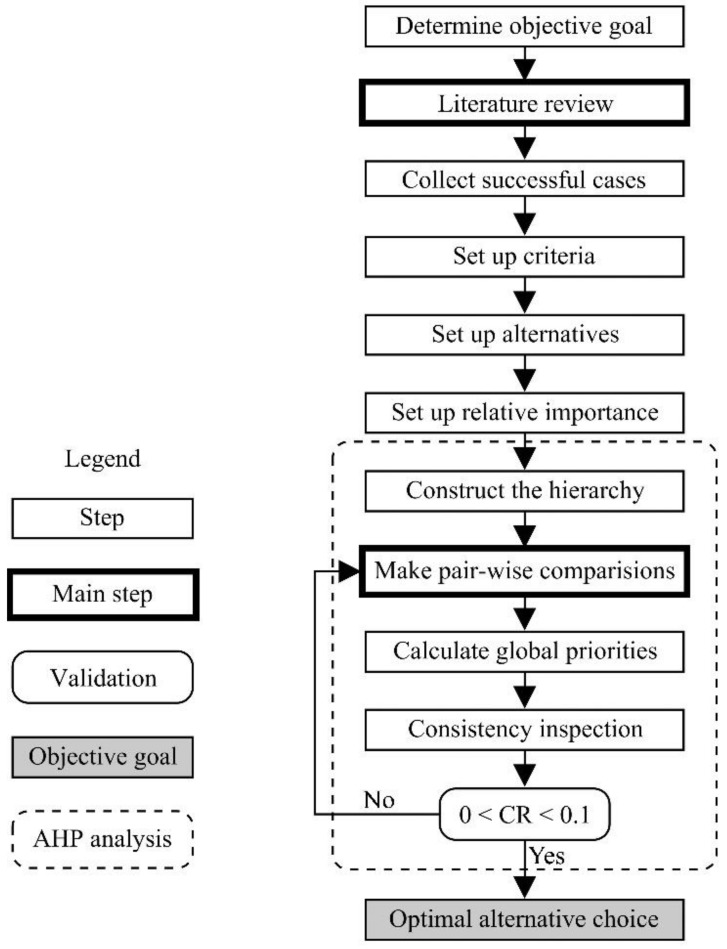
Decision-making model design. Source: Prepared by the authors.

Priorities (P) represent the relative weights of the nodes in any level hierarchy. ‘Weight’ can refer to importance (or preference, or likelihood) or whatever factor is being considered by decision making process (i.e., goal—N1—, criteria or influencing factors—N2—, and alternatives or initiatives—N3—). The priorities of each hierarchical level—local priorities—always add up to 1.00 (100%). Regarding our analysis, the sum of the alternative priorities at each criterion level adds to 1.00 (100%). The global priorities (i.e., the priority of each alternative at global level—GP—) are obtained by adding the values resulting from multiplying the alternative priority at each criterion level by the criterion priority. These alternative global priorities add to 1.00 (100%). Each GP represents the importance (i.e. weight) of its corresponding alternative in the analysis as a whole. Thus, the global priority of alternatives means a key factor to decide which of them seems to be the most successful facing urban decline in the case study. The decision-making model design is shown in Figure 1.

#### 3.3.2. AHP Analysis

The information obtained in the previous steps was processed using an AHP multi-criteria matrix. Pair-wise comparisons of criteria (N2) and alternatives (N3) were made according to the Saaty’s numerical scale (1–9), highlighting the relative importance of some elements over others in each hierarchical level [30]. Alternatives level pair-wise comparison was carried out according to the defined criteria. The following comparative scale values are given: 1 = the same importance; 3 = weak dominance; 5 = strong dominance; 7 = demonstrated or very strong dominance; 9 = absolute dominance. In addition, the coherence of the model was checked by calculating the Consistency Index (CI), the matrix being consistent when CI < 10%. The results of the pair-wise comparisons are arranged in a matrix. The first (dominant) normalized right Eigen vector of the matrix gives the ratio scale (weighting) [30]. The Eigen value $\lambda_{max}\;$ determines the Consistency Index: $CI=\frac{\lambda_{max}-n}{n-1}$ where n is the comparison matrix size. The AHP analysis was performed using the web tool AHP Online System (https://bpmsg.com/ahp-online-system/).

It should be noted that the design and structuring of an analytical hierarchy ‘is more an art than a science’ since there is not a precise expression for the identification or stratification of the elements involved in the process [43]. For this reason, an attempt was made to design the decision-making model based on logical criteria, such as: (i) dominance type in pair-wise comparisons of both criteria and alternatives; (ii) information, priorities and frequencies obtained from a literature review; or (iii) emergence and applicability level of initiative profiles. For the assignment of relative importance in each hierarchical level it is commonly used an expert panel, although there is evidence of studies supported by literature reviews regarding the study field [44,45], a methodological approach followed in this work.

The input data (attributes of successful cases facing urban decline) were collected from mostly theoretical academic studies, which are the most common in this field (see Table 2). In general, these studies do not handle large volumes of objective data produced by agreed indicators that allow empirically contrasting the strategies developed against urban decline in each case. However, they do usually include general information such as population data, goals stated, and different factors that led to involve some recovery strategies rather than others. For this reason, the AHP multi-criteria analysis was considered more suitable for assessing the different alternatives, as compared to other DSS such as decision trees (DT) from data mining computational field, despite its apparent similarity. 

Briefly, AHP is a structured quantitative and qualitative method based on multiple criteria of experts’ judgement that are pair-wise compared to obtain the priorities of several alternatives [46]. In contrast, DT is a quantitative technique initially more suitable for massive data analysis in which decision alternatives are divided according to probabilities and prefixed rules until the most likely one is found [47]. Both methods are useful for solving complex problems. In both cases the representation looks like a hierarchical tree, but using a different processing algorithm. A limitation of the AHP method is that subjective factors cannot be completely excluded [48]. To avoid this, an expert agreement is often taken into account, which in this work was replaced by a literature review (which in a sense is the same thing). This also makes it possible to identify objective criteria and relative importance for the criteria and for the alternatives according to each criterion. Both methods can be used together in the same analysis. For example, the AHP method would set priorities, and the DT method would show the sequential path of all possible decisions up to the most suitable alternative [49].

#### 3.3.3. Relative Importance of Criteria and Alternatives

Given the exploratory approach of the method and in order to develop a more realistic study, the assessment of relative importance (RI) is carried out using a numerical range (1–5) of pair-wise comparison, excluding values 7 (demonstrated dominances) and 9 (absolute dominances). The values adopted at each hierarchical level are justified as follows:

(i) Criteria pair-wise comparison (C1–C4). In general, the most important thing is to strategically plan the initiative type according to the cause of the problem and the goal to be achieved in each case, and that it is also feasible. In addition, the measure scale is a factor to be taken into account, even though it is known that the same initiative can be applied in a multi-scale manner [50,51]. Therefore, at a first level of importance, an RI = 5 is provided for the initiative profile (C3), a criterion associated to the strategy against the decline developed in each case. A second level of importance is considered using RI = 3 based on decline origin (C2) and initiative cost (C4). Finally, at a third level of importance, RI = 1 is provided for the population-size range criterion of each city studied (C1), understood as a more relative decision-making factor. Regarding the results, this last choice encourages that the most suitable alternatives to the case study do not depend so much on the population-size factor as on other higher relative importance criteria. 

(ii) Alternatives pair-wise comparison (1–12). (a) For population-size range (C1) and decline origin (C2) criteria, the relative importance of each alternative (i.e., initiative) is assigned according to its similarity with the case study (RI = 3 for similar cases; RI = 1 for other cases). The aim is to prioritize those initiatives that are comparatively developed in a contextual framework close to the case study, which may help to ensure the strategic suitability of the selected initiatives. (b) According to the literature review, and within the SDG framework of the UN Agenda 2030, the sustainable initiatives facing urban decline, and especially the environmental ones, seem to be the most emerging and frequently successfully applied. This type on initiatives, even those that keep an industrial use but ecologically adapted, are the ones that better preserve the environmental and natural base of a territory, being therefore the most valuable for sustainability [52,53]. On the other hand, environmental initiatives of ecological nature tend to be the lowest cost [54]. Hence, an RI = 3 is assumed for environmentally sustainable measures (being chosen RI = 1 for others) within the initiative profile criterion (C3). (c) Finally, regarding the cost factor, priority is given to the lowest cost alternative, i.e., those with the highest viability (RI = 5) as compared to those with a medium (RI = 3) or high cost (RI = 1). Each alternative is weighted by giving these RI values, obtaining a hierarchical list of initiatives against decline (A–L; n = 12) according to the case study.

## 4. Results

Through a literature review, general information on successful cases facing urban decline were collected and detailed in Table 2, at least in some respects, such as (a) landscape measures on post-industrial footprint in Cleveland and Philadelphia, showing an increase in close properties values [3]; (b) environmental mitigation and ecological restoration by means of the ‘industrial forests’ project in the Ruhr Valley [41,42]; (c) resizing infrastructure or ‘flexible’ transport in New York [38]; (d) densified urban ‘archipelagos’ proposal in Berlin [35]; (e) community oriented planning to stop population loss in St. Louis [5]; (f) incentive policies for birth rate, subsidy and capital in Western European countries since the middle of the 20th century; (g) recovery initiatives based on attracting creative social class according to the Creative Cities project [55]; or (h) central city renewal projects of large Latin American metropolitan areas (e.g., São Paulo, Buenos Aires or Mexico DF) with a view to keeping population in these areas, despite their intrinsic problems (e.g., gentrification, overcrowding, insecurity), and reaching the global city status [36,39,40], among others. In addition, some successful initiatives in the USA are based on a public-private relationship. With this, vacant land left by the earlier production system (Fordism) which demands rehabilitation, land decontamination or landscape measures, is transferred in exploitation to private property which is done in exchange for investing to recover them with new land uses (Post-Fordism). This investment may be horizontal (e.g., the implementation of parks and playgrounds in New York, or golf courses in Chicago’s Downtown); or vertical by redensification interventions using mixed uses, such as in Baltimore or Houston [34].

Table 3 shows the different cases collected grouped into alternatives when the cases are similar (A–L) and classified according to the criteria adopted (C1–C4), as well as the relative importance assigned to each of them (1–5). This is the base table where the different hierarchical levels and their processing are justified, with a view to the subsequent AHP analysis. According to the literature reviewed, there is a dominance of alternatives focused on large cities (75%), with a decline origin of economic base (75%), which developed measures facing the decline of different profile (33% each case) and high cost (42%). This bias should not influence the analysis results since the sample was weighted according to the relative importance assigned at each hierarchical level. Due to its emergency, the absence of cases with a decline origin based on environmental factors is noteworthy. However, the UN and other institutions have been talking about ‘climate refugees’ for years, though their legal status has not yet been recognized.

**Figure 2:**
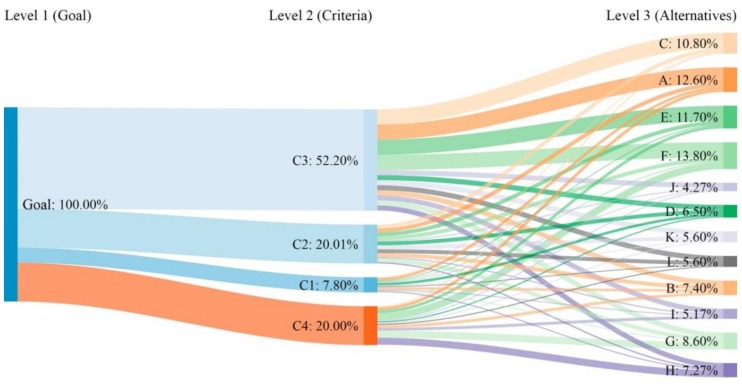
Priorities (%) of the elements of each hierarchical level. Notes: (1) The sum of criteria and alternatives = 100% in both cases. (2) The flow width from the criteria to the alternatives shows the weight of each criterion in each alternative. Source: Prepared by the authors (Sankey diagram).

**Figure 3:**
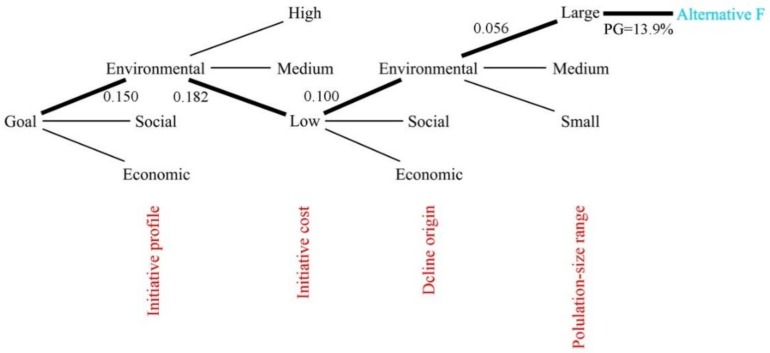
The decision tree structure constructed for the decision process. Notes: The criteria used in the sequential decision tree were sorted according to priorities obtained by the AHP structure. Local priorities are shown along the branches in each step. Global priority for alternative F (the one that best suits the case study) is given as a percentage. The criteria are shown in red. Due to spatial limitations only one branch of each criterion was shown. Source: Prepared by the authors.

The results of the AHP analysis are shown in Table 4, and the AHP workflow in Figure 2. In addition, in order to clarify the decision route to the most suitable alternative, the associated sequential decision tree was represented schematically (Figure 3). In this scheme, the rest of branches were omitted to avoid a too extensive figure. According to the case study (Mieres) and the literature reviewed, the most suitable alternative is F (cases 17, 18, 25, i.e., New York–New York/Chicago–Ruhr Valley; PG = 13.9%). It is formed by large cities (see Table 2) that had problems in their productive economic sectors, and that tried to solve them using dominantly environmental and low-cost initiatives (see Table 3). On the other hand, the least suitable alternative to the case study is J (cases 10, 14, i.e., Halle/Leipzig–México DF; PG = 4.3%), which is formed by large cities showing a decline origin of social base, and that faced it using high-cost economic profile measures. Between these two extremes, the different alternatives show several combinations of the mentioned factors. 

The similarity of the cities grouped under the alternative A (1, 12, 22, i.e., Avilés–Lieksa–Puertollano; PG = 12.7%) to the case study in terms of population-size range (medium-sized) and decline origin (economic) should be noted. In addition, these cities adopted environmental correcting measures of medium cost. However, it ranks second in terms of applicability to the case study, behind alternative F which has different features.

## 5. Discussion

This work followed the research line of some authors such as Großmann et al., Hollander et al. and Sánchez-Moral et al. [5,14,16] and meetings (e.g., UC Berkeley–SCiRN, 2007) that call for more international and comparative studies that collect lessons learned from urban decline cases in an integrated way, which would be useful in new cases where this phenomenon is still active. In this line, in order to explore which successful cases against shrinkage, grouped under alternatives, would be more suitable to the case study (Mieres), an AHP analysis was carried out. This method is not intended as a substitute but rather as complementary to other assessment methods of already proved validity (e.g., surveys, direct observations, expert panels). Most of the cases collected in Table 2 are located in the northern hemisphere (92%), in line with Oswalt and Rieniets [6]. The complexity of the problem, due to the existence of several hierarchical levels (i.e., multiple alternatives dependent as well on several criteria of variable influence), demands the support of this useful tool for decision-making in planning [30,43,56].

Based on a literature review, in order to refine the results, several criteria of different influence were identified, prioritizing alternatives oriented towards sustainability (dominantly environmental and low-cost for the reasons argued along the text), following the steps of other works such as Hemphill et al. and Lee and Lim [31,32]. In this line, regarding the decision-making model, the penalization or even exclusion of high-cost alternatives could seem obvious without the need to develop a multi-criteria analysis. Nevertheless, as the assessment is multidimensional and therefore complex, it becomes less evident. Logically, the results showed coherent coincidences, such as the case study (Mieres) and the optimal alternative (F—New York–New York/Chicago–Ruhr Valley—) had the same decline origin (economic-industrial), which brings one closer to the other. However, there were some apparent divergences between them in the results (e.g. the population-size range factor), which were not intuited in the early methodological steps until the results were obtained. This showed how the alternative most likely to be successful if applied to the case study involves large cities. This, a priori, might seem inconsistent. However, are the largest population-size range cities those that drive the talent, creativity, resources and innovation necessary to carry out sustainable recovery initiatives more efficiently [57]. Thus, the measures successfully developed in them might be extended to lower urban categories by local adaptation. 

The finding of this work does not lie in the full coincidence between the attributes of the case study and those of the alternative most likely to be applied successfully facing the urban decline of the former, but in the fact that measures against decline that involve sustainable and low-cost initiatives could be multi-scale reproduced regardless of factors such as the decline origin of the population-size range. For this reason, the optimal alternative is not A (1, 12, 22, i.e., Avilés–Lieksa–Puertollano; PG = 12.7%), apparently more similar to the case study, but F (17, 18, 25, i.e., New York–New York/Chicago–Ruhr Valley; PG = 13.9%) (see Table 3 and Table 2—alternatives as groups of similar cities/regions initiatives coded by numbers are shown in Table 3. The links between numbers, i.e. cases, and cities/regions are identified in Table 2; see case collection column—). Even so, the results are not intended to be unique or exclusive but exploratory and indicative based on the arguments and steps taken. 

### 5.1. Integration of the AHP Method Together with Data Mining Tools

Lines of future research could improve the accuracy of the analysis model by increasing, for example, the case collection number, detailing the available information, increasing the number of influencing criteria, and probably performing and additional multi-criteria analysis at the micro-level (i.e., between the case study and the optimal alternatives). These measures would significantly increase the volume of data. Therefore, the integration of AHP and data mining techniques would be useful to process the information and detail the most suitable alternatives. As an example of this, based on the study of Liu and Shih on customer lifetime and products recommendation [58], and on the study of Xi et al. on traffic accident causation [59], the integrated analysis would proceed as follows: (i) obtaining global priorities using an AHP method; (ii) profiling alternatives of similar priorities using a clustering algorithm; and (iii) using association rule learning (i.e., a rule-based machine learning method) to identify probabilities of dominant trends in criteria associations taking into account global priorities. 

The last two steps would proceed as follows, considering the research of Agrawal et al. and the previous authors [58,59,60]. Alternatives with similar GPs according to weighted criteria would be grouped using a clustering algorithm (e.g., k-means). The factors determining the alternatives form a set of elements I. The set of possible associations of I is called D. So that the rule X⇒Y is followed, where X,Y⊆I and X∩Y=0. Condition X (antecedent) being the possible associations of criteria and subcriteria; and Y (consequent) being the similar GP alternative clusters. In order to find the most interesting associations of I, restrictions can be set based on minimum support and confidence thresholds, where:

$$supp\left(x\right)=\frac{\left|X\right|}{\left|D\right|}\;\text{and}\;conf\left(X\Rightarrow Y\right)=\;\frac{supp\left(X\cap Y\right)}{supp\left(X\right)}=\frac{\left|X\cap Y\right|}{\left|X\right|}$$

This analysis would allow to know the more frequent criteria-subcriteria association in each, for example, high, medium or low global priority cluster of alternatives within a set formed by multiple cases facing urban decline. The most interesting would be the high GP associations (desired replicability) and low GP associations (avoided replicability). This would help to identify frequent successful and/or failure patterns according to specific case studies, while also removing the least influential factors and thus reducing analysis complexity, which can help decision-making against urban decline. Additionally, there is evidence of geographic information systems (GIS) as a useful tool for the objective information collection step (case collection in this work) from spatial databases. Later, this information can be weighted using an AHP analysis to prioritize decisions, such as Narimisa and Namirisa did in their work on environmental impact assessment of the introduction of a new industrial land use [61]. 

As noted above, the global priority determines the alternative that best suits the case study. As shown in Figure 2, this priority depends on the relevance of each alternative according to each criterion, which is shown by the width of the associated flow between criteria and alternatives. Likewise, the criteria priorities (local priorities) are different from each other. Note how the ‘winner’ alternative F receives the maximum possible flow (i.e., it is assessed with the highest relative importance along the pair-wise comparison in each case) from almost all criteria (see Figure 2 and Table 4). The results from the AHP method were integrated into a sequential decision tree to facilitate understanding of the decision process as for example Suner et al. did in their medical research [49]. 

### 5.2. Implementation of Alternatives Facing Urban Decline Using the Future Scenarios Tool

In Mieres, some measures were already taken under alternative A (Avilés–Lieksa–Puertollano), such as investment in renewable energies (thermoelectric, solar), but they have not yet meant definitive measures against the decline of this city today. Nevertheless, the initiatives encouraged by alternative F (New York–New York/Chicago–Ruhr Valley) have not yet been explored in this city. No other type A measures, compatible with those of type F as explained below, were taken in Mieres either. At this point, the optimal alternatives facing the decline would be clear, but how can they be captured in a specific case study?

Knowing and detailing the most suitable alternatives facing urban decline according to a specific case study is one thing, but facilitating and guiding their implementation in that case study is another. The latter can be a challenge due to high uncertainty, wide range of future states and complex interdependencies this process generates [62]. It would produce an urban transition state that should be informed to the different stakeholders to avoid social conflicts and to anticipate market potentials [63]. The construction and prospective assessment of possible urban scenarios can be useful for this transition [64,65,66]. To get an in-depth view from them, the approach and assessment should be multidimensional. Some key factors of this process can be (i) the evaluation indicators choice; (ii) the diversity of scenarios; (iii) the stakeholder opinions; and (iv) the type of assessment analysis. Some key evaluation indicators may include desirability, usefulness and probability [67]. This work argued for a sustainable urban transition based on the assessment of alternatives against decline. It is a type of transition widely supported by the literature on urban transition planning, which in turn involves shrinking cities [50,68]. The evaluation of the most suitable alternatives through the construction of future scenarios would make it possible to know, for example, stakeholders preferences regarding the most sustainable or less sustainable scenarios, whether the most preferred scenarios match the more or less probable ones, or whether there are differences between the evaluation of the different stakeholders. 

Following the indications of Bügl et al. and Majoor, the construction of diverse future scenarios can be done based (i) on their systematic variability concerning sustainability, dominant criterion in the work approach; and (ii) on different urban areas (e.g., family, luxury, failure and transitory districts) [67,69]. To consider decision-making the structure of the scenarios should be made according to different design components of sustainable planning (environmental, social and economic), such as social environment, eco-design, building, financing, and social infrastructure. In the case study, these design components should be detailed based on measures collected by one or more of the highest global priority (GP) alternatives (such as F and A alternatives), according to Table 4 and Table 2, and the basic information collected in Table 1, which can be extended in future research.

Another option for constructing future scenarios would be based, for example, on the spatial approach of Kropp and Lein [70]. For this purpose, the case site would first have to be mapped and evaluated quantitatively before and after the impact caused by the various level improvements from the highest GP alternative. Objective indicators of environmental, social and economic sustainability would be used for the assessment, such as pollution prevention, community connectivity degree, brownfield redevelopment degree, access to public transport, parking capacity, habitat restoration suitability, amount of vacant land, or heat island effect, among others. These indicators would be weighted according to their relative importance using a multi-criteria decision-making method (MCDM), such as AHP analysis. Finally, the mapping of the different scenarios would be done based on the priority given to each sustainability component through their respective indicators. This method would allow prioritizing the environmental component, connecting with the dominant profile of the highest GP alternative of this work. 

Both for the design of sustainable scenarios based on the measures provided by the priority alternatives [67,69], and for the mapping and evaluation of their impact [70], the case study shows the following potentials, among others: (i) vacant urban and peri-urban post-industrial land; (ii) 6 linear km of radial riverside pedestrian paths length; (iii) 200 linear km of greenways surrounding the city (old mining railway lines); (iv) existence of expectant industrial heritage; and (v) commercial land use linked to pedestrian streets within the central urban area. For information only, these potentials suggest the following visions to be evaluated: (a) the ‘industrial forests’ project along the Emscher River in the Ruhr Valley occupying case study brownfields [41,42]; (b) the development of new economic activities such as forestry and logging [19]; (c) new interconnected urban green areas along with affordable land available for alternative land use options [2,5,24,25] with public-private financing and use [34], and flexibly communicated [38] that would provide the city with identity and new economic drivers [71,72,73].

## 6. Conclusions

The utility of collecting successful experiences facing urban decline and applying a DSS-AHP analysis to explore at the macro-level which would be the most suitable measures for a particular case study was evidenced. This made it possible to prioritise certain criteria and alternatives over others, which was an aim of the work. In this process, the literature review was used as a valuable complementary method to the expert panel for the relative importance assignment at different hierarchical levels. Low-cost environmental initiatives seemed to be the most sustainable and suitable to the case study, regardless of other a priori important factors such as the decline origin or the population-size range, which was a novelty. 

Analysing and implementing measures against urban decline in a particular shrinking city, which have already been successfully developed in other cities, can be a challenge due to the complexity and uncertainty of the process. The integration of various decision-support systems may be useful for it. In this work, the convenience of weighting the impact of the different alternatives in the case study from a sustainable approach was evidenced using a multi-criteria assessment. Subsequently, the decision tree representation clarified the decision route until the optimal alternative facing the decline was reached. In order to improve the model, in the event of increasing information, the usefulness of clustering algorithms and associations rules learning was discussed with a view to identify patters of frequent factors based on global priorities, which may optimise the decision-making process. Finally, the value of constructing future urban scenarios as an additional decision-making tool for implementing the desired alternatives in the case study was also discussed. In this process, having detailed information on the profile of the suitable alternatives and on the case study attributes can help to decide and characterise both the different scenarios and their design components. 

According to the analysis carried out, the most suitable alternative facing urban decline in the case study (GP = 13.9%) involves infrastructural flexibility (as was previously done in New York), public-private investment and exploitation for new urban uses in vacant land (as in New York/Chicago), and environmental mitigation and ecological restoration through planting and use of forests (as in Ruhr Valley). This group of measures linked economic development with sustainable planning, which is desirable given the attributes of the case study. However, according to the assessment done and the baseline information data, the case study showed significant limitations (GP = 4.3%) for the development of measures based on massive public subsidies and systematic building demolitions (as in Halle/Leipzig), and urban renewal of central urban areas supported by large-scale interventions (as in Mexico DF). These are high-cost measures that cannot be assumed by a medium population-size range and a compact central urban fabric that is not attractive enough to capture large amounts of capital. In addition, they would not take advantage of the reuse potential of certain historic urban fabric, post-industrial land uses, and heritage elements found in the city. Intermediate impact measures (GP = 7.3%) of low-cost social origin and profile such as the use of a governance focused on the social capital accumulation (as in Estonia/Central Germany), local social initiatives and the development of subsistence agriculture (as in Ivanovo), and a planning leading to social cohesion (as in St. Louis) may improve the quality of life but miss the environmental potential of the case study as the main driver of development (vacant land, partial network of walkable green areas between the main city and dispersed populations, etc.). Furthermore, it would force synergies of social nature that traditionally were not developed in a particular form to mean a contemporary incentive. 

## Figures and Tables

**Table 1:** Case study summary table.

Scope	Weaknesses/Threats	Strengths/Opportunities
Non-residential land uses	Flood plains used for old industrial uses	Obsolescence of the industrial fabric
	Old industrial uses next to residential uses	Obsolescence of the industrial fabric
	Obsolete urban fabric	Demolitions, reuse
	The commercial use shows some development potential within the urban centre, but this area is deficient	Pedestrianisation of some streets in the city centre
	Scarce tourism	Existence of a rich industrial heritage
Residential land uses	40% of the population employed in Mieres resides outside Mieres	-
	Obsolete urban fabric, lack of housing	Demolitions, reuse (recovery of old and central mining neighbourhoods through public funding)
	Failed new housing developments (including social housing –VPO–)	-
Heritage	Facade interventions only, abandoned or poorly built elements	Elements of historical industrial interest
	Recovery of old railway lines as greenways (approx. 200 km), but only existing around the urban area	Post-industrial land available in urban areas
Infrastructure	High-speed train (AVE) not available	The aim is to get an AVE stop on the León-Gijón line.
	Dominant transport by private vehicle, very few km of cycling routes	Municipal bus lines available, fluvial pedestrian walkways of interest available
Economy	Non-competitive traditional mining-steel activity	Alternative economic sectors under development (e.g., thermoelectric energy, solar energy, renewed steel industry, building materials, ICTs, tertiarization, services)
	Hard to attract new economic sectors despite new incentives	-
	Excess of public funding	-
Environment	Pollution (derived from thermoelectric and cement plants, among others)	Natural environment
Others	General lack of urban land	Obsolescence of the industrial fabric

Source: Prepared by the authors based on the research of Tomé [10].

**Table 2:** Some successful city cases against urban decline.

Case Collection	Declining Sector	Reference	Dominant Recovery Strategies	Initiative Profile
1. Avilés (SP)	Economic (steel and iron industry)	[11,16]	Urban tourism, functional and landscape transformation of brownfields, environmental adaptation, historic centre and port regeneration	Environmental
2. Baltimore/Houston (US)	Economic (industrial)	[34]	Post-industrial public land given in exploitation to private property in exchange for investment and new vertical uses	Economic
3. Berlin (GE)	Economic (industrial)	[35]	Urban densification policies	Social
4. Bilbao (SP)	Economic (steel and iron industry)	[9]	Industrial restructuring, urban revitalisation, new urban facilities and services (metro, new urban nodes, etc.), tourism	Environmental
5. Asturian mining cities (e.g. Mieres, Langreo) (SP)	Economic (mining, steel and iron industry)	[10]	Adaptation, reindustrialization, tertiarization, urban transformation	Economic
6. Cleveland (US)	Economic (industrial)	[3,5,34]	Landscape transformation of the post-industrial footprint	Environmental
7. Detroit (US)	Social (racial, social, spatial segregation)	[13]	Urban transformation of central areas, cultural and creative revitalization of suburbs, guided immigration	Social
8. Estonia/Central Germany (ES/GE)	Social (political, post-socialism, economic restructuring)	[22]	Governance focused on the accumulation of local social capital	Social
9. Fuxin (CH)	Economic (lack of resources)	[33]	Experimental structural economic change (settlement of technology parks and economic development areas in general)	Economic
10. Halle/Leipzig (GE)	Social (emigration due to German reunification)	[2,13,28]	Public subsidies, mass demolition operations	Economic
11. Ivanovo (RU)	Social (USSR’s fall, globalization, deindustrialization	[13]	Subsistence agriculture, post-industrial practices, local social initiatives	Social
12. Lieksa (FI)	Economic (industrial –natural resources processing–)	[19]	Resilience and adaptability based on: wood industrial sector transformation, especially nature tourism, and internet and phone (call-centres) economy	Environmental
13. Manchester/Liverpool (UK)	Economic (industrial) and social (insecurity, unemployment)	[13]	Recovery of empty buildings in central urban areas, new urban culture (music, fashion, media), public-private partnerships, call-centre development	Social
14. México DF (central city) (ME)	Social (gentrification, insecurity, emigration)	[8,36,37]	Urban renewal of the historic centre through large-scale investments (walkways, high-rise buildings, singular projects)	Economic
15. Mulhouse/Roubaix/Saint-Etienne (FR)	Economic (industrial –steel and iron, textile, weapons–)	[21]	Creative talent attraction and social mix to drive urban economic growth	Social
16. Newcastle (UK)	Economic (shipyards)	[33]	Transformation into a museum, arts and sciences city centre	Social
17. New York (US)	Economic (industrial)	[38]	Infrastructure resizing, flexible transport	Environmental
18. New York/Chicago (US)	Economic (industrial)	[34]	Post-industrial public land transferred in exploitation to private property in exchange for investment and horizontal uses	Environmental
19. Philadelphia (US)	Economic (industrial)	[5,34]	Landscape transformation of the post-industrial footprint	Environmental
20. Pittsburgh (US)	Economic (steel and iron industry)	[3,5]	Settlement of prestigious universities and research centres	Social
21. Ponferrada (SP)	Economic (mining, industrial)	[10]	Industrial investments	Economic
22. Puertollano (SP)	Economic (mining, industrial)	[10]	Industrial adaptation to renewable energy, green tourism, CO_2_ reduction	Environmental
23. São Paulo (central city) (BR)	Social (gentrification, overcrowding, inequity)	[36,39,40]	City centre renewal through the social reuse (cultural, major events) of historic buildings with public-private investments	Social
24. St. Louis (US)	Social (emigration)	[5,34]	Community and social cohesion oriented urban planning	Social
25. Ruhr Valley (GE)	Economic (industrial)	[41,42]	Environmental mitigation and ecological restoration by planting post-industrial forests	Environmental

Source: Prepared by the authors based on their literature review.

**Table 3:** Influencing factors (criteria C1–C4), initiatives (alternatives A–L), and relative importance (1–5).

Alternatives (Cities/Regions)	C1 (1) Population			C2 (3) Decline Origin			C3 (5) Initiative Profile			C4 (3) Initiative Cost		
	LA (1)	MD (3)	SM (1)	EN (1)	SO (1)	EC (3)	EN (3)	SO (1)	EC (1)	HI (1)	ME (3)	LO (5)
A (1, 12, 22)		●				●	●				●	
B (5)		●				●			●		●	
C (4)	●					●	●			●		
D (21)		●				●			●	●		
E (6, 19)	●					●	●				●	
F (17, 18, 25)	●					●	●					●
G (3, 13, 15)	●					●		●				●
H (8, 11, 24)	●				●			●				●
I (7, 23)	●				●			●			●	
J (10, 14)	●				●				●	●		
K (16, 20)	●					●		●		●		
L (2, 9)	●					●			●	●		

Legend: LA: Large; MD: Medium-sized; SM: Small; EN: Environmental; SO: Social; EC: Economic; HI: High; ME: Medium; LO: Low; (n): relative importance (1–5). To know the cities that form each alternative (A–L) see Table 2 (case collection column). Source: Prepared by the authors based on literature review.

**Table 4:** Decision-making hierarchy.

N1	N2 (Criteria)		N3 (Alternatives)											
Goal	Cr	GP%	A	B	C	D	E	F	G	H	I	J	K	L
**Suitable initiative according to the case study**	C1	7.8	0.167	0.167	0.056	0.167	0.056	0.056	0.056	0.056	0.056	0.056	0.056	0.056
	C2	20.0	0.100	0.100	0.100	0.100	0.100	0.100	0.100	0.033	0.033	0.033	0.100	0.100
	C3	52.2	0.150	0.050	0.150	0.050	0.150	0.150	0.050	0.050	0.050	0.050	0.050	0.050
	C4	20.0	0.077	0.077	0.030	0.030	0.077	0.182	0.182	0.182	0.077	0.030	0.030	0.030
	**GP%**	**100.0**	**12.7**	**7.4**	**10.9**	**6.5**	**11.8**	**13.9**	**8.7**	**7.3**	**5.2**	**4.3**	**5.6**	**5.6**

Notes: In all cases the decision matrix is robust (CI < 2%); Cr: Criterion; GP: Global priority. Source: Prepared by the authors based on the AHP model results.
